# Repeated Glutathione Sodium Salt Infusion May Counteract Contrast-Associated Acute Kidney Injury Occurrence in ST-Elevation Myocardial Infarction Patients Undergoing Primary PCI: A Randomized Subgroup Analysis of the GSH 2014 Trial

**DOI:** 10.3390/life13061391

**Published:** 2023-06-14

**Authors:** Alessio Arrivi, Giacomo Pucci, Martina Sordi, Marcello Dominici, Francesco Barillà, Roberto Carnevale, Amalia Morgantini, Riccardo Rosati, Enrico Mangieri, Gaetano Tanzilli

**Affiliations:** 1 Interventional Cardiology Unit, “Santa Maria” University Hospital, 05100 Terni, Italy; martinasordi@yahoo.it (M.S.); prof.dominici@gmail.com (M.D.); amaliamorgantini@gmail.com (A.M.); riccardorosatimd@gmail.com (R.R.); 2 Unit of Internal Medicine, “Santa Maria” University Hospital, 05100 Terni, Italy; giacomo.pucci@gmail.com; 3 Department of Medicine and Surgery, University of Perugia, 06123 Perugia, Italy; 4 Department of Systems Medicine, University Tor Vergata, 00133 Rome, Italy; francesco.barilla@uniroma2.it; 5 Department of Medical-Surgical Sciences and Biotechnologies, Sapienza University, 04100 Latina, Italy; roberto.carnevale@uniroma1.it; 6 IRCCS Neuromed, Località Camerelle, 86077 Pozzilli, Italy; 7 Department of Clinical, Internal Medicine, Anesthesiology and Cardiovascular Sciences, Sapienza University of Rome, 00161 Rome, Italy; enrico.mangieri@uniroma1.it (E.M.); gaetano.tanzilli@uniroma1.it (G.T.)

**Keywords:** glutathione, contrast-associated acute kidney injury, STEMI, primary percutaneous coronary intervention

## Abstract

Background: Contrast-associated acute kidney injury (CA-AKI) is still a major concern for referring physicians, especially in the setting of ST-elevation myocardial infarction (STEMI) patients undergoing primary-PCI (pPCI). To evaluate whether glutathione sodium salt (GSS) infusion impacts favorably on CA-AKI, an unplanned exploratory data analysis of the GSH 2014 trial was performed. Methods: One hundred patients with STEMI were assigned at random to an experimental group (No. 50) or to a placebo group (No. 50). Treatment consisted of an intravenous infusion of GSS lasting over 10 min before p-PCI. The placebo group received the same quantity of normal saline solution. After the interventions, glutathione was administered in the same doses to both groups at 24, 48 and 72 h. Results: CA-AKI occurred in 5 out of 50 patients (10%) allocated to the experimental group (GSS infusion) and in 19 out of 50 patients (38%) allocated to the placebo group (*p* between groups < 0.001). No patients in either group required renal replacement therapy. After allowing for multiple confounders, GSS administration (OR 0.17, 95% CI 0.04–0.61) and door-to-balloon time (in hours) (OR 1.61, 95% CI 1.01–2.58) have been the only independent predictors of CA-AKI. Conclusions: the results of this sub-study, which show a significant trend towards an improved nephroprotection in the experimental group, led to the hypothesis of a possible new prophylactic approach to counteract CA-AKI using repeated GSS infusion. Subsequent studies with specific clinical outcomes would be necessary to confirm these data.

## 1. Introduction

Contrast-associated acute kidney injury (CA-AKI) is a possible cause of acute renal dysfunction in hospitalized patients, which also occurs in those undergoing primary PCI (pPCI) due to ST-elevation myocardial infarction (STEMI) [1,2]. It is defined by a decline in renal function that is expressed by an increase in serum creatinine of more than 25% or 44 μmol/L from baseline within 3 days of the intravascular administration of iodinate contrast media (CM) agents [3]. It may develop on either a pre-existing failure of renal function or a normal function [4] and is often associated with a poor prognosis [5]: temporary dialysis may be necessary in up to 15% of patients [6] and in those without full renal recovery there is a risk of progression towards chronic and/or terminal renal disease [7]. Protracted hospitalization, as well as an increased probability of illness and/or death, are associated with its occurrence [8]. Age, door-to-balloon time, troponin-T peak value, female sex, type 2 diabetes (T2DM) and contrast volume to estimated glomerular filtration rate (eGFR) ratio are independent risk factors for its development [9]. Its pathophysiology is not entirely known; the main theories are that it is caused by kidney ischemia, oxidative insult and direct injury to tubular epithelial cells [10,11]. To date, there is still no endorsed treatment for this disease once it is present, making prevention the best option. However, no consensus defines the most successful intervention to counteract CA-AKI; guidelines still recommend the intravenous administration of isotonic saline or sodium bicarbonate in patients at increased risk of this disease [12]. The potential positive impact of antioxidants on CA-AKI is currently under investigation. In animal models of acute renal failure, an increased concentration of reduced glutathione (GSH) has been shown to be associated with an attenuated rise in creatinine [13]. However, its potential role in humans has not been researched to date. An unplanned exploratory analysis of the data collected in a randomized, placebo-controlled clinical study (the GSH 2014 trial) was conducted [14] to gain knowledge of the potential role of GSS infusion in preventing CA-AKI development.

## 2. Methods

Details of the GSH 2014 trial were reported in the main study [14]. Basically, consecutive patients with STEMI, over 18 years old and referred to the three p-PCI HUB centers, were selected for enrollment in the GSH 2014 trial (EudraCT number 2014-004486-25). Exclusion criteria were the following: door-to-balloon time > 12 h, rescue PCI, critical hypotension (systolic blood pressure < 90 mmHg) secondary to cardiogenic shock, left main involvement, angiographic evidence of coronary collateral vessels (Rentrop score of 2 or 3 for the area under consideration), scars following a previous myocardial infarction, saphenous venous graft disease, severe renal impairment with an eGFR less than 30 mL/min/1.73 m^2^, acute infection, treatment with systemic corticosteroids or oral anticoagulants, malignancy, in-stent thrombosis, absence of consent to participate. The study was planned according to principles of the Declaration of Helsinki. AIFA authorization and single ethical committee acceptance was obtained by all three centers. The protocol was made by the coordinating center. An external core lab handled the data. Having been informed and given consent, 100 patients were assigned in a 1:1 random manner to the experimental group (No. 50), or to the placebo group (No. 50), respectively. Treatment was based on an intravenous infusion of GSS (2500 mg/25 mL, Biomedica Foscama Group, Rome, Italy), lasting over 10 min before p-PCI. The placebo group received the same amount of a sodium chloride 0.9% (normal saline) solution. Patients underwent p-PCI according to the standard protocols. The choice of arterial access (radial or femoral) was left to the discretion of the operator. Once the aortic lumen was reached with angiographic guidance, a bolus of 70 IU/kg of unfractionated heparin was administered intravenously. Possible supplements were added in order to maintain an activated clotting time of ≥250 s during the procedure. Coronary angiography was carried out according to conventional standards: at least four views (right anterior oblique (RAO) Caudal, RAO Cranial, left anterior oblique (LAO) Cranial and LAO Caudal (the spider one)) for the left coronary artery and two views (LAO and LAO Cranial) for the right coronary artery. The same type of nonionic, low-osmolarity-contrast agent was used to perform the angiography at each of the three centers (Iomeron^®^ 350 mg/mL, Bracco Imaging Italia srl, Milan, Italy). All patients were treated with drug-eluting stents implant in the coronary vessels. After the interventions, GSS was administered at the same doses at 24, 48 and 72 h. Random allocation of patients to intravenous infusion of GSH or placebo (saline solution) was performed prior to pPCI by means of computer-developed series code assignment. The physicians who performed the p-PCI, those who carried out post-hoc analysis of the digital angiograms, and the laboratory staff were not informed of study-treatment allocation. Blood chemistry and bio-humoral data were collected at the same time intervals for each patient: a venipuncture from an antecubital vein of the arm was carried out immediately before the start of the angioplasty and repeated daily for the following three days. CA-AKI was diagnosed as an increase in serum creatinine over 25% or 44 μmol × L^−1^ from baseline within 3 days of the intravascular administration of iodinate CM agents, as per the definition [3].

## 3. Statistical Analysis

The assumption of normal distribution of continuous variables was tested using the Kolmogorov–Smirnov test. Comparisons between groups were performed using the Student’s *t*-test for parametric variables and Kruskal–Wallis test for non-parametric variables. A stepwise multivariate logistic regression was used to analyze the independent predictors of CA-AKI development in the presence of other potentially explanatory variables. In order to achieve this, randomization allocation, age, female sex, T2DM, baseline eGFR, door to balloon time, procedural time, troponin-T peak value, contrast volume to eGFR ratio, number of stents, rates of anterior STEMI, Killip class ≥ 3, atrial fibrillation, previous PCI and statin use were all introduced in the multivariate logistic regression model. Non-parametric variables were all logarithmically transformed before entering the model. The software used for statistical analysis was Statistical Package for Social Sciences (SPSS) version 26. A *p* value below 0.05 was considered statistically significant.

## 4. Results

**Figure 1:**
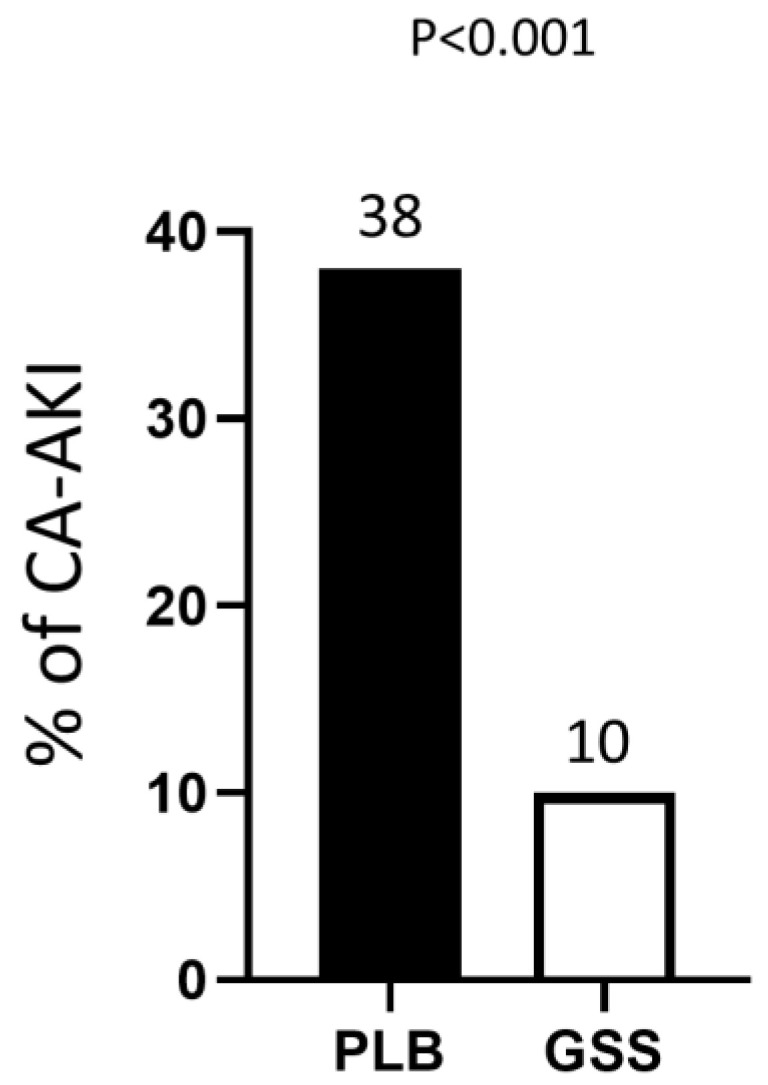
Proportion of contrast-associated acute kidney injury (CA-AKI) diagnosed among patients randomized to placebo (PLB) or glutathione sodium salt (GSS) infusion.

CA-AKI was diagnosed in 5 out of 50 patients (10%) allocated to the experimental group (GSS infusion) and in 19 out of 50 patients (38%) allocated to the placebo group (*p* between groups < 0.001) (Figure 1). No patient in either group required renal replacement therapy. Patients developing CA-AKI did not substantially differ from patients who did not develop CA-AKI in all the examined variables, although non-significant trends toward older age, lower BMI and T2DM prevalence, higher female proportion, higher troponin peak and baseline eGFR, as well as longer door-to-balloon time, were observed (Table 1). After adjustment for multiple confounders, GSS administration (OR 0.17, 95% CI 0.04–0.61) and door-to-balloon time (in hours) (OR 1.61, 95% CI 1.01–2.58) were the only variables associated with the outcome measure. The remaining variables were not closely associated with CA-AKI development.

## 5. Discussion

The main purpose of the present sub-analysis was to evaluate the potentiality of repeated GSS infusion to favorably impact the occurrence of CA-AKI in STEMI patients undergoing p-PCI. The results of this investigation, which show a significant trend towards an improved nephroprotection in the experimental group, confirm our hypothesis. Oxidative stress induced by ROS and vasoconstriction have been implicated in the etiology of CA-AKI [15]. ROS may lead to direct tubular and vascular damage, thus exacerbating renal parenchymal hypoxia presenting early after exposure to the CM [16]. The reperfusion injury, following STEMI, may further expand the initial injury through an ongoing process of apoptosis and inflammation that takes place over hours or days [17]. GSH is a water-soluble tripeptide; it works directly, by capturing reactive oxygen and nitrogen species, or indirectly, by increasing enzymatic activity as a cofactor [18]. GSH is able to pass through the mitochondrial membrane, and is stored in the endoplasmic reticulum [19]. Starting from our initial hypothesis [20], we clearly demonstrated in our pilot trial that the administration of GSS in patients with STEMI was followed by the significant depletion of H_2_O_2_ production and an increase in NO bioavailability [21]. The latter finding was associated with a 21% reduction in myocardial injury, resulting from a significant drop in serum cardiac troponin T (cTpT) release during the 5 days of reperfusion [21] and reduced hospitalization [22]. Furthermore, in a randomized subgroup analysis of the same trial (GSH2014) [23], we demonstrated a less steep increase in serum NO bioavailability in patients who developed contrast-mediated nephropathy than in the control group, thus highlighting a possible critical role of NO depletion in the pathogenesis of the nephropathy. According to the above results, we hold that both GSS scavenging action on the free radicals and the exacerbated vasodilation following the increase in the NO bioavailability may counteract CA-AKI development. The reduction in effective blood volume and the consequent hypoperfusion resulting from this reduction in the cardiac output following STEMI may play an additional role in the determination of renal damage (the so-called cardio-renal mechanism) [15,24]. In addition, advanced age, female gender, diabetes and contrast volume have been used as further triggers [9]. The fact is that, in our analysis, the two groups were found to be well balanced both in terms of anthropometric and procedural characteristics. This made it possible to limit the bias related to confounders, giving more value to the result. Only increased door-to-balloon time was found to be positively correlated (OR1.61) with the development of CA-AKI, although this result was expected according to the recent literature [25]. Furthermore, we believe that the administration of GSS, not only as a single dose but also repeated over the three days following the pPCI, was of value in counteracting the oxidative burst, which may be prolonged [17]. This would justify the concept of the prolonged administration of the antioxidant, which was well adapted to our study, considering the ease of preparation and administration of GSS as well as the lack of significant side-effects [26]. The latter is of relevance when compared to other widely used antioxidants, such as N-Acetylcysteine (NAC). It may be noted that important adverse effects, such as anaphylactoid reactions, were found in up to 8.2% of patients after the parenteral administration of the NAC [27], and its clinical efficacy was not fully demonstrated, as the existing literature shows inconsistent results [28]. Additionally, supplementation with vitamin C was investigated as a preventive treatment against CA-AKI in patients undergoing coronary angiographic procedures, although doubts still persist regarding its dosage and bioavailability [29]. Even vitamin E, when added to an adequate hydration, has shown interesting preliminary nephroprotective results, which need to be confirmed in further studies [30]. Therefore, we believe that both the biomolecular characteristics of GSH itself [19] and the proposed methodology (whole administration of 2500 mg, not as a single bolus, but repeated in the 72 h following the reopening of the culprit vessel) [22] can explain the above results. In fact, a sub-dosage of GSH and as a single administration has not shown nephron-protective effects in patients undergoing coronary angiography to date, even in the setting of an elective, non-emergent procedure [31]. The context of an acute event, such as STEMI, is different: the inflammatory response secondary to the massive release of ROS, vasoconstriction, leukocyte recruitment and infiltration at the level of the injured parenchyma compromise renal function through tubular and microvascular damage [32]. In particular, the presence of kidney CD206+ (detected with immunohistochemical techniques) was found to be correlated to the impairment of renal function and early death [33]. It is probably in this scenario that the scavenging and NO-releasing action of GSH can make the best contribution to the preservation of renal function. Moreover, the use of the GSS allows us to overcome problems related to cardiac preload control during pPCI, considering the unfavorable impact of a saline overload on a left ventricle with a reduced output due to myocardial infarction [24]. Rapid intravenous administration over 10 min before the start of the coronary angiography allows for the immediate achievement of the drug’s steady state [24] and a prompt and lasting antioxidant action (considering the repetition of the administration) following the reopening of the infarct-related coronary vessel. Last but not least, the low cost of the GSS must be taken into account [34], resulting in less public healthcare spending. The prevention of CA-AKI through the use of GSH has important consequences as it can favorably impact the duration of hospital stay [22], improving patient turnover, and thus also preventing complications associated with prolonged hospitalization [35]. In conclusion, we can state that the prophylactic approach against CA-AKI using repeated GSS infusion (immediately before and in the days following the pPCI) may be considered as a possible method to counteract this undesired eventuality. Further studies with larger sample size are necessary to confirm these preliminary results. 

## 6. Limitations

The sample size (100 patients) of this sub-analysis is too small to draw definitive conclusions; some clinical data are missing (e.g., blood pressure at admission and rate of intraprocedural hypotension, as well as details of 14–30 days of renal function follow-up). Morover, even if the randomized treatment allocation and the double-blind design guaranteed a comparable adjustment of the possible causes of CA-AKI between groups, the GSH 2014 trial was not powered for clinical outcomes. Therefore, the results still have to be validated in additional studies.

## Figures and Tables

**Table 1:** Main characteristics of the study population. BMI: body mass index, T2DM: type 2 diabetes mellitus, eGFR: estimated glomerular filtration rate.

	CA-AKI	No CA-AKI	*p*
N,	24	76	
Male sex, %	65	73	0.50
Age, years	69 (10)	68 (11)	0.78
BMI, kg/m^2^	25.1 (4)	26.8 (4)	0.11
T2DM, %	37	38	0.94
Peak troponin, ng/mL	193 [102–200]	178 [178–207]	0.66
Door to balloon, hh:mm	4:40 (1:35)	3:57 (1:23)	0.06
Baseline eGFR, mL/min/1.73 m^2^	86 [66–93]	81 [66–94]	0.66
Contrast volume/eGFR ratio,	2.68 [2.31–3.04]	2.64 [2.22–3.31]	0.84
Anterior STEMI, n (%)	8 (33)	34 (45)	0.32
Killip class ≥ 3, n (%)	2 (9)	3 (4)	0.40
Atrial fibrillation, n (%)	2 (8)	9 (12)	0.63
Previous PCI, n (%)	2 (8)	3 (4)	0.40
N. of stents,	1.2 (0.3)	1.2 (0.4)	0.95
Procedural time, min	54 (15)	54 (13)	0.98
Statin use, n (%)	13 (54)	34 (45)	0.49

## Data Availability

The data presented in this study are available on request from the corresponding author.
